# Neurological Disease Rises from Ocean to Bring Model for Human Epilepsy to Life

**DOI:** 10.3390/toxins2071646

**Published:** 2010-06-28

**Authors:** John S. Ramsdell

**Affiliations:** Marine Biotoxins Program, Center for Coastal Environmental Health and Biomolecular Research, NOAA, National Ocean Service, Charleston, SC 29414, USA; Email: john.ramsdell@noaa.gov; Tel.: +1-843-762-8510; Fax: +1-843-762-8700

**Keywords:** domoic acid, harmful algal bloom, dichlorodiphenyltrichloroethane (DDT), sea lion, epilepsy

## Abstract

Domoic acid of macroalgal origin was used for traditional and medicinal purposes in Japan and largely forgotten until its rediscovery in diatoms that poisoned 107 people after consumption of contaminated mussels. The more severely poisoned victims had seizures and/or amnesia and four died; however, one survivor unexpectedly developed temporal lobe epilepsy (TLE) a year after the event. Nearly a decade later, several thousand sea lions have stranded on California beaches with neurological symptoms. Analysis of the animals stranded over an eight year period indicated five clusters of acute neurological poisoning; however, nearly a quarter have stranded individually outside these events with clinical signs of a chronic neurological syndrome similar to TLE. These poisonings are not limited to sea lions, which serve as readily observed sentinels for other marine animals that strand during domoic acid poisoning events, including several species of dolphin and whales. Acute domoic acid poisoning is five-times more prominent in adult female sea lions as a result of the proximity of their year-round breeding grounds to major domoic acid bloom events. The chronic neurological syndrome, on the other hand, is more prevalent in young animals, with many potentially poisoned *in utero*. The sea lion rookeries of the Channel Islands are at the crossroads of domoic acid producing harmful algal blooms and a huge industrial discharge site for dichlorodiphenyltrichloroethane (DDTs). Studies in experimental animals suggest that chronic poisoning observed in immature sea lions may result from a spatial and temporal coincidence of DDTs and domoic acid during early life stages. Emergence of an epilepsy syndrome from the ocean brings a human epilepsy model to life and provides unexpected insights into interaction with legacy contaminants and expression of disease at different life stages.

## 1. Primer of Domoic Acid Toxicity

**Figure 1:**
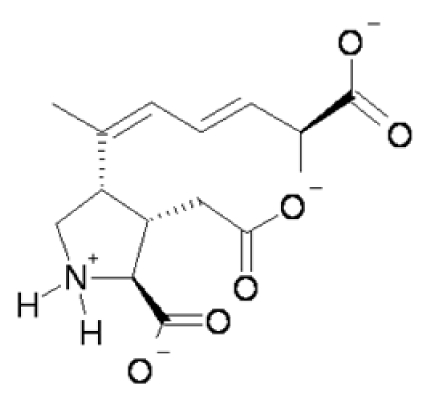
Predicted predominant charge form of domoic acid at physiological pH.

The subject of domoic acid has recently been reviewed from pharmacologic, neuropathogenic and neurobehavioral perspectives by several investigators [1,2,3]. This section is designed to call attention to the basic elements of domoic acid toxicity. The information is updated from a detailed review of the molecular and integrative basis of domoic acid toxicity which may be referenced for detailed information and citations [4]. Domoic acid is a tricarboxylic acid with a unique large carboxylic side chain (Figure 1). At physiological pH the predominant species of domoic acid is deprotonated at all three carboxyl groups and protonated at the amino group leading to a net charge of negative 2.

**Figure 2:**
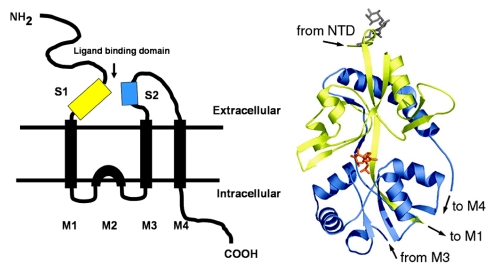
Left: Schematic of ionotropic gluatamatergic receptor with S1 and S2 ligand binding domains highlighted in yellow and blue, respectively. Right: Minimized structure of GluR6 S1-S2 fusion protein with domoic acid shown as an orange stick figure bound between the S1 and S2 domains. Reprinted with permission from National Academy of Sciences, USA [5].

The degree of protonation of domoic acid limits its oral toxicity to animals to well over parts per thousand exposure levels. The low pKa of domoic favors its rapid absorption as a neutral acid in the stomach; however, overall absorption of an oral dose is very low. Upon entering the bloodstream, domoic acid has a very short half life as a result of rapid renal filtration. Domoic acid poorly penetrates most regions of the central nervous system and it gains primary access though circumventricular organs. In regions of the brain where capillary endothelial cells restrict the entry of domoic acid, the toxin slowly transverses endothelial cell membranes by pinocytosis. Once domoic acid reaches its neuronal target tissue, it binds with high affinity to kainate subtypes of ionotropic glutamate receptors, with a functional unit containing three transmembrane spans and one intracellular membrane span (Figure 2, left). The ionotropic receptor is comprised of homomers assembled into two pairs of dimers yielding a tetrameric structure. Functional channels in the brain are usually a mixture of two different homomers, with the KA1 and KA2 subunits requiring association with members of the GluR5–7 family. Domoic acid binds to a site that includes both the first (S1) and second (S2) extracellular domain (Figure 2 right). 

**Figure 3:**
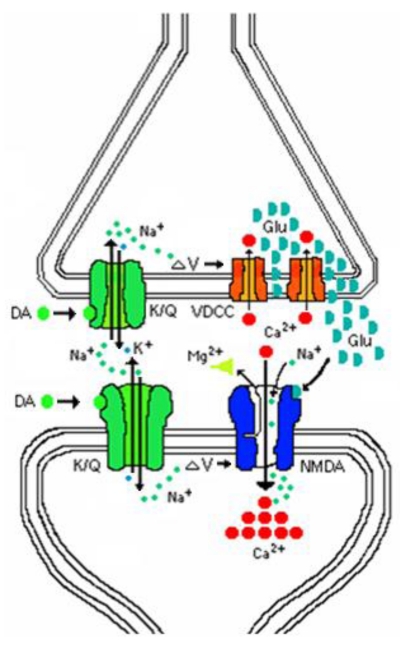
Schematic of pre- and postsynaptic actions of domoic acid.

Domoic acid affects only a partial opening of the channel and it interferes with the closing, resulting in a sustained ion conduction that yields a net influx well beyond that of natural neurotransmitters. This level of excitotoxicity is reached from a coordinated action on both sides of the synapse (Figure 3). Domoic acid activates both presynaptic and postsynaptic kainate receptors leading to a sodium ion (Na^+^) influx and membrane depolarization. The presynaptic depolarization activates voltage gated calcium channels and elevates intracellular calcium (Ca^2+^), releasing glutamate into the synapse. Postsynaptic depolarization releases the magnesium (Mg^2+^) block of the NMDA receptor and glutamate activates the receptors causing a massive calcium flux, and thus triggering excitotoxicity. 

**Figure 4:**
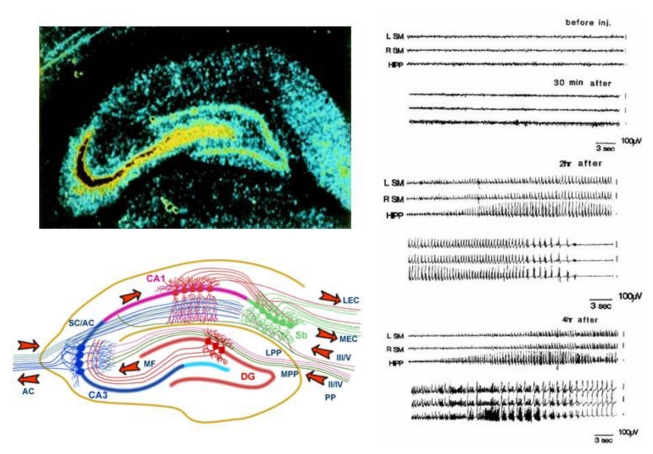
Top left: Autoradiogram of kainic acid binding sites in hippocampus. Reprinted with permission from Elsevier [6]. Bottom left: Afferent and efferent pathways of the hippocampus. Reprinted with permission from MRC Centre for Synaptic Plasticity, University of Bristol, (2005) [7]. Right: Domoic acid effects on EEG activity in rats. Reprinted with permission from Elsevier [8].

The huge concentration of kainic acid receptors in the CA3 region targets domoic acid to the hippocampus (Figure 4, top left) where its action overrides a signaling pathway normally propagated through closed loop, seizure-prone circuitry in the medial temporal lobe of the brain (Figure 4, bottom left). Uncontrolled electrical activity (seizures) (Figure 4 right) is outwardly observed as contraction and relaxing of muscles (convulsions) and an acute episode of these seizures is referred to as status epilepticus (SE).

**Figure 5:**
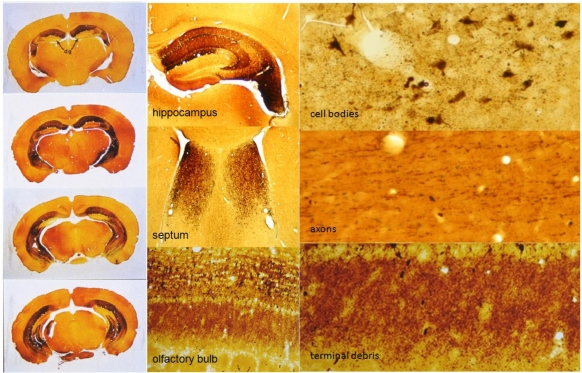
Domoic acid damage to brain assessed by cupric-silver histochemistry and viewed at three levels of magnification. Domoic acid degenerated neuronal elements are stained black via cupric-silver degeneration stain which is an advantageous method because it can be viewed at three levels of organization (full coronal section, specific brain regions and cellular detail). Degenerated pathways at the full coronal section can been seen throughout the ammon’s horn of the hippocampus (left) and limbic regions of the hippocampus, septum and olfactory bulb (center). At the cellular level, affected cell bodies appear as large black regions normally associated with terminal debris Axons appear as thin filamentous strands and were typically distal to the regions where labeled cell bodies appeared. Degenerated terminals are identified as small pinpoints, often associated with the cell bodies.

Permanent effects of domoic acid include brain damage in the form of neuronal loss and astrocytosis in the hippocampus which can spread throughout the limbic regions of the brain (Figure 5), as well as deficits in spatial learning and memory.

## 2. Rare Natural Product to Novel Shellfish Poison

**Figure 6:**
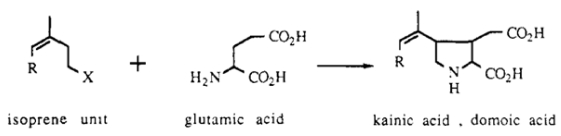
Biosynthetic scheme for domoic acid.

Domoic acid and the structurally related compound kainic acid were discovered in several red macroalgal (*Rhodophyta*) seaweeds that had been used for traditional medicine since the ninth century in Japan [9,10]. These unique chemical structures have been proposed to be analogs of the neurotransmitter *L*-glutamate condensed with one or two isoprene units to form the side chain. Fusion of the first isoprene unit with the amino group forms the pyrrolidine ring (Figure 6) [11]. Subsequent isotopic enrichment studies have supported this biosynthetic origin and further indicated that alpha-ketoglutarate may serve as the source of glutamate for biosynthesis [12]. Kainic acid, isolated from a more plentiful seaweed, quickly gained recognition as a potent excitotoxin and found popular use as an experimental tool and model agent for epilepsy research [13,14,15]. Domoic acid, isolated from a rarer source of seaweed, was sparsely investigated until it was briefly examined for its strong insecticidal activity [16] three years in advance of its recognition as the causative agent of a human outbreak of neurotoxic poisoning in Montreal, Canada [17,18].

**Figure 7:**
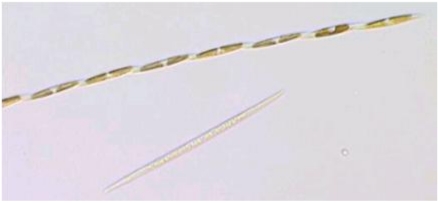
Light micrograph of *Pseudo-nitzchia multiseries*. Reprinted with permission from Dr. S.L. Morton [22].

In this widespread poisoning, the victims had consumed blue mussels (*Mytilus edulis*) harvested from Cardigan Bay in Prince Edwards Island that were contaminated with the diatom *Pseudo-nitzchia multiseries* (Figure 7) [19]. An unusually dry summer in 1987 followed by a wet fall caused terrestrial run off of inorganic silicate, which is believed to have sustained a massive bloom of *P. multiseries* for three winter months. Periodic depletion of silicate by the growing cells appears to have stressed the cells and prompted them to produce domoic acid [20]. Huge concentrations of the toxin, measured as high as 790 ppm, accumulated in the mussels; high enough to poison more than 100 individuals and severely poison 14 individuals of advanced age or renal impairment [21].

Whereas the macroalgal source of domoic acid *doumoi* (*Chondria armata)* was known as efficacious on the Ryukyu island of Tokunoshima for ring worm diseaseand on the more northerly island of Yakushima to control flies, consumption of the toxin contaminated mussels revealed a new sequel of gastrointestinal and sustained neurological symptoms in the 107 confirmed cases. Nineteen percent of the cases were sufficiently severe to merit hospitalization (half in intensive care units) and ultimately led to four deaths which occurred between one and 12 weeks. Eight percent of all patients had episodes of status epilepticus, which completely resolved after four hours to 12 weeks. One quarter of all patients suffered a loss of short term memory, some of which was permanent. Neuropsychological testing of a subset (n = 14) of severely poisoned individuals, 4–6 months after the event, identified a disproportionate deficit in memory compared to other cognitive functions, with a primary effect on delayed recall of verbal and visuospatial material [23]. Hence, domoic acid poisoning from consumption of contaminated mussels was clinically recognized as amnesic shellfish poisoning (ASP) [24]. Domoic acid has subsequently been found globally in shellfish, therefore a regulatory limit of 20 ppm for recreational and commercial harvests has been imposed. It has reached 230 ppm in Washington razor clams [25] and staggering levels of 2900 ppm in the viscera of Irish scallops [26]. However, human poisoning has not been reported outside the 1987 event in Canada. 

## 3. Human Epilepsy Model Comes to Life

Nearly a year after the Canadian ASP event, an 84 year old male survivor re-experienced severe seizures [27]. This individual had initially experienced disorientation the day after eating the contaminanted mussels and by the third day focal seizures had progressed to status epilepticus. Seizures were nonresponsive to the mild anticonvulsant phenytoin and responded only to large doses of phenobarbitol. The electroencephalograms (EEG) showed electrogenic abnormalities. Although structural abnormalities were not evident by brain computed tomographic scan, it is possible that current MRI techniques may have resolved abnormalities to the hippocampus. Seizures were controlled by phenobarbitol and he was discharged from the hospital seizure-free four and half months after the poisoning. He continued with phenobarbitol management for two additional months and had a normal EEG at eight months. One year after the acute poisoning, the man exhibited an acute episode of focal seizures with clonic contraction of the left arm and leg. These seizures responded to phenytoin and were controlled with continuing pharmacotherapy. One and a half years later, the individual developed pneumonia and died. The autopsy revealed bilateral hippocampal sclerosis. 

This single case provided observational evidence that domoic acid induced status epilepticus can progress after a latent period to temporal lobe epilepsy (TLE) as a result of mesial temporal sclerosis. However, it was of interest to epilepsy research in that it provided a real case scenario for an important experimental model for human TLE. The kainic acid model of experimental epilepsy has yielded a detailed understanding of some of the processes involved in human acquired epilepsy. Acquired, or “injury-induced” epilepsy, accounts for about half of human TLE and can be caused by status epilepticus, febrile seizures, stroke or head injury [28]. When administered systemically or intracerebroventricularly, kainic acid induces status epilepticus and produces an epilepsy syndrome similar to human TLE [15]. This model has three stages: (1) an initial toxic insult manifests as a several hour episode of status epilepticus; (2) a period of “silent toxicity” normally spanning several weeks; and (3) the development of a permanent state of spontaneous recurrent seizures (SRS): the hallmark of the epileptic state [29]. Although kainic acid acts via specific endogenous kainic acid receptors to produce the neurological sequelae [30], this model has been critiqued because the inducing agent is a neurotoxin. This single domoic acid human case study therefore provided an unexpected “real life” context in which to view the kainic acid model [27].

**Figure 8:**
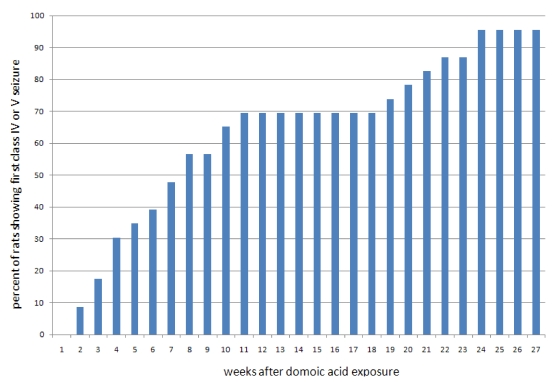
Onset of seizures after a single domoic acid induced status epilepticus.

Perez-Mendes and colleagues subsequently designed a study to evaluate the suitability of domoic acid for a model of chronic temporal lobe epilepsy (TLE) [31]. Systemic administration of domoic acid to marmosets (*Callithrix jacchus*) induced status epilepticus; however, the doses of domoic acid required to induce status epilepticus proved lethal. Additional attempts to limit the status epilepticus period to five minutes by “anticonvulsant rescue” with diazepam followed by intensive care and monitoring of animals proved insufficient to prevent death. Furthermore, lower doses of domoic acid which caused minor convulsive behavior and long term survival caused no recurrent seizure behavior over six months of monitoring. Therefore, domoic acid was reported not to be effective for the generation of a model of chronic temporal lobe epilepsy in nonhuman primates. However, domoic acid induced status epilepticus in rats has recently been found to reproduce SRS when administered in a way that limits its acute lethality [32]. Titration of the administered doses has proven to be an effective means to reduce lethality of kainic acid and maintain a high outcome of SRS. Dudek and co-workers developed an hourly low-dose systemic exposure protocol for kainic acid in rats that promotes long term survivability and a high success rate of inducing an epileptic state [33]. Based on this research, administration of a short series of hourly low doses using domoic acid also induces a nonlethal status epilepticus that is sufficient after a mean latent period of 5 weeks to induce a permanent epileptic state (Figure 8) [32]. Furthermore, domoic acid does so with high efficiency such that greater than 90% of animals exhibit SRS within six months. Hence domoic acid, like kainic acid, can be used as a test compound to understand the aspects of acquired TLE. 

Not all TLE is associated with status epilepticus nor is evidence of a precipitating CNS injury. Up to half of human TLE cases are idiopathic: they are presumed genetic and are likely to originate during development with symptoms appearing at defined ages [34]. Interestingly, the same neurotransmitter systems involved in excitotoxicity in adult animals are operational for different purposes during development [35] and lead to different epileptic states as described further in this manuscript. For example, titration of subconvulsive domoic acid doses daily for the second postnatal week manifests later in life as persistent effects, which include changes in emotionality, inducing low grade seizures and increasing seizure sensitivity and stereotypic behaviors [36]. Thus, domoic acid has served as a test agent to produce a developmental model for epilepsy [37], and hence may serve both as a test agent for idiopathic as well as acquired forms of epilepsy. The above described kainic acid and domoic acid experimental models, along with the single human case study, are now applicable to the hundreds of sea lion cases, which represent a sentinel population poisoned by domoic acid with rich information of the disease state during different life stages. 

## 4. The Northern Anchovy Summons Sentinels of Domoic Acid Poisoning

**Figure 9:**
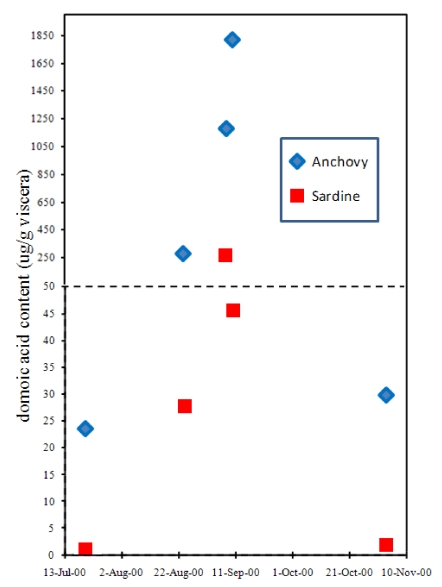
Accumulation of domoic acid in anchovies and sardines sampled at the same site in Monterey Bay, CA. Chart is plotted with data from Lefebvre *et al.* [40].

Although the accumulation of domoic acid in blue mussels led to its recognition as a natural toxin that severely poisons humans, another vector has proven far more lethal, at least to wildlife. The northern anchovy (*Engraulis mordax*) is abundant in the surface waters of the California current and Monterey Bay and is a highly efficient filter feeder of another toxigenic *Pseudo-nitzchia* species, *P. australis.* Toxin levels in the anchovy average ten-fold higher concentrations than its nearest competitor, the Pacific sardine (*Sardinops sagax*) (Figure 9) [38,39,40]. Because very little (0.2%) of the toxin that accumulates in the gut of the anchovy is absorbed into body tissues, this species can transfer high toxin loads to predators [39]. As a combined result of efficiency to accumulate *Pseudo-nitzchia* and safe compartmentalization of toxin burden, the anchovy is a deadly vector for domoic acid that can intensify relatively minor blooms of *P. australis* into major mortality events of marine animals [39,41].

The origin of wildlife domoic acid poisoning can be traced to a 1961 article in the *Santa Cruz Sentinel*, documenting unusual behavior of hundreds of sooty shearwaters (*Puffinus griseus*) in the coastal town of Capitola, CA [42]. According to residents, the birds crashed into buildings disgorging fish, showed aggressive behavior and a shrieking vocalization uncommon for this species [42]. The newspaper was contacted by filmmaker Alfred Hitchcock who used this report as part of his film adaption of the Daphne du Maurier novelette *The Birds*. Retrospectively, this unusual bird behavior has been suggested to be the result of domoic acid poisoning. Years later, unexplained mortality events of the brown pelican (*Pelecanus occidentalis*) were common on the central California coast, with major events recorded in fall of 1971, 1976 and 1981 [43]. Then in September 1991, a mass mortality of brown pelicans and Brant’s cormorants (*Phalacrocorax penicillatus*) was observed in Monterey Bay, with rescued animals exhibiting stereotypic scratching, a characteristic behavior response to domoic acid previously observed in mice treated with the mussel extract from the Canadian ASP event [44]. Upon further investigation, the birds’ stomachs were determined to be filled with anchovies containing domoic acid and frustules of *P. australis* [45]. 

The California sea lion is often viewed as the primary target species for domoic acid poisoning; yet, it can also be viewed as a sentinel for the marine environment. Although the term “sentinel” has been used interchangeably with “indicator”, this review adheres to the O’Brien *et al.* [46] interpretation of these similar terms. From this perspective, an indicator species alerts to the presence of environmental conditions that are not as readily measured for another species or the environment as a whole; whereas sentinels show changes in characteristics that can be measured to determine the extent of environmental contamination and impact for health of other species of concern. 

Mortality events of sea lions with signs of neurological poisoning were recorded in California in 1978, 1986, 1988 and 1992 and neuropathology of animals from the 1992 event was recognized years later as consistent with domoic acid poisoning [47]. The defining event occurred in the late spring of 1998. Over 400 sea lions were found stranded dead from Monterey Bay to San Diego and at least 70 surviving animals were observed to exhibit typical domoic acid symptoms of ataxia, seizures and coma [41,48]. The poisoning was correlated with a late spring bloom of *P. australis,* contaminated anchovies, and domoic acid was found in the urine of affected sea lions [41].

The poisoning of sea lions has been a consistent sentinel of the impact of domoic acid events for other marine species that inhabit waters along the California coast. For example, sea lions engage in cooperative foraging behavior with common dolphins along the ridges and canyons of the Southern California Bight [49]. Indeed long and short-beaked common dolphins (*Delphinus capensis* and *D. delphis*) are often found stranded with sea lions [50]. Late spring schools of anchovies have been observed to summon multi-species feeding activity including humpback whales feeding with hundreds of dolphins and sea lions and thousands of sooty shearwaters [51]. At least eleven other cetacean species found stranded have been confirmed poisoned with domoic acid remaining in feces or urine. These other cetaceans include Dall’s porpoise *(Phocoenoides dalli)*, harbor porpoise *(Phocoena phocoena)*, bottlenose dolphin (*Tursiops truncatus*), Risso’s dolphin *(Grampus griseus)*, Northern right whale dolphin *(Lissodelphis borealis)*, Pacific white-sided dolphin *(Lagenorhynchus obliquidens)*, humpback whale *(Megaptera novaeangliae)*, blue whale *(Balaenoptera musculus)*, fin whale *(Balaenoptera physalus)*, northern minke whale (*Balaenoptera acutorostrata*), and sperm whale *(Physeter macrocephalus)* [52]). The levels of domoic acid measured in cetaceans can be very high and porpoises, dolphins and whales have been found with levels exceeding 4 µg/g in feces and urine, a level rarely found in sea lions [52]. The number of stranded cetaceans is small compared to the number of sea lions stranded, as cetaceans are less likely to survive seizures to strand onshore [50]. Yet, the sea lion can serve as a sentinel for cetaceans, with the caveat that the differences in brain structure and physiology of the animals may not fully predict observable symptoms [53]. Additionally, the sea lion is more amenable to long term clinical evaluation to follow the progression of domoic acid poisoning to neurological disease.

## 5. Neurological Disease on the Rise in California Sea Lions

**Figure 10:**
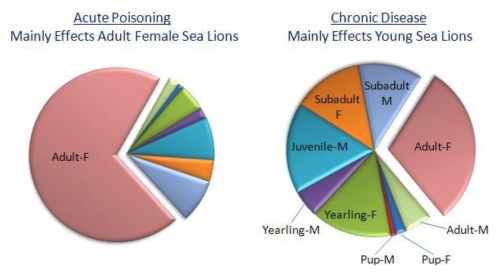
Age distribution of acute (left) and chronic (right) cases of neurological disease associated with domoic acid poisoning of sea lions. Chart is plotted with data from Goldstein *et al.* [55].

The stranding of sea lions exhibiting epileptic seizures has occurred along the California coast nearly every year since 1998, with five clusters of acute poisonings recorded [54,55]. The first event in the spring of 1998 centered in Monterey Bay and appears to have spread as far south as San Diego county [47]. The other four occurred in mid to late summer of 2000, 2001, 2002 and 2005 and were centered off the coast of San Luis Obispo and Santa Barbara counties. Acute domoic acid poisoning events have been defined by the following criteria: at least five cases admitted into rehabilitation over 48 hours, exhibition of a continuous state of seizures and/or ataxia for up to a week’s duration, stranding clustered within 80 km of coastline, and hippocampal necrosis identified in at least one case [48,55]. The number of overall acute cases fluctuated in each of these years without showing an increasing trend. Adult female sea lions represent the majority of cases, exceeding other age groups several fold (Figure 10, left chart).

Clinical and epidemiological analyses of stranded sea lions admitted to the Marine Mammal Center (Sausalito, CA) over an eight year period have revealed a second clinical presentation besides acute poisoning. Of 715 sea lions stranding with neurological symptoms, nearly one quarter of the animals did not fit the above described criteria for acute poisoning events. These animals stranded individually, expressed intermittent seizures and unusual behaviors, with individual strandings peaking approximately four months after an acute poisoning event. Therefore, these were designated as chronic neurological cases. Clinically, 112 of the chronic neurological cases were observed to develop intermittent seizures varying in duration from hours to weeks, which is characteristic of seizure induced brain damage. Another 32 animals treated for acute poisoning developed intermittent seizures after a seizure free period ranging from two to twelve weeks. Another six animals initially admitted for acute poisoning were readmitted after release with intermittent seizures and undetectable levels of domoic acid. The seizures observed were generalized from simple or focal seizures to tonic-clonic seizures, with the clonic phase commonly showing extension of flippers and the neck, and often resulting in a convulsive status epilepticus. These seizures were pharmacoresistant to several anticonvulsants, including phenobarbital, and the majority of the animals died or were euthanized due to poor prognosis. Magnetic resonance imaging (MRI) identified structural abnormalities which were described by neurohistology as primary hippocampal damage spreading through the limbic system. This chronic disease is largely represented by younger sea lions of both sexes (Figure 10). 

The preponderance of adult female sea lions acutely poisoned by domoic acid reflects their year round occupancy at rookeries that are positioned in the reach of frequent toxic *Pseudo-nitzschia* blooms. By contrast, males spend only a short period at the rookeries in June to breed before migration out of the region heavily impacted by these blooms. Given a gestation period of nearly a year, and mating each summer other than during periods of lactation, female sea lions spend much of their adult life span pregnant. This contributes to the extensive amount of reproductive failure seen in California sea lions in the last decade that has been associated with toxic *Pseudo-nitzschia* blooms [41,55,57]. The 1998 event occurred during the last month of gestation and of the 54 adult females admitted for rehabilitation, nearly one-third experienced prenatal reproductive failure [58]. An even larger event in 2002, which also occurred just prior to term of parturition, resulted in 209 documented cases of domoic acid associated reproductive failure [56]. These results indicate that not only are pregnant females poisoned by domoic acid but most likely their fetuses are poisoned as well. Indeed, domoic acid has been found in the amniotic fluid of sea lions [56], common dolphins and harbor porpoises [52]. Experimental studies in laboratory rats have shown the toxin to be readily transferred across the placenta to sustain high levels in fetal plasma and collect in amniotic fluid [57]. However, reproductive poisoning of this population of sea lions and their fetuses has been observed for at least 25 years and has been attributed to other causes as described in the next section.

## 6. Toxic Legacy of a Post WWII Industrial Period

**Figure 11:**
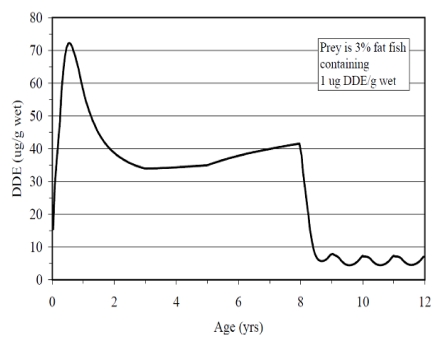
*p,p’*-DDE toxicokinetics in the California sea lion modeled on consumption of contaminated fish. Reprinted with permission from Elsevier [64].

The organochlorine pesticide DDT (dichlorodiphenyltrichloroethane, used here to refer to all DDT related compounds) is a persistent pollutant that was banned in the U.S. in the early 1970s. An estimated 100 tons remains in sediments of the Palos Verdes shelf in southern California due to more than twenty years of industrial and agricultural discharge [60,61]. The transfer of DDT from sediments to the biota of the foodweb, along with the reproductive failure of the brown pelican and bald eagle on the neighboring Channel Islands [62,63], triggered monitoring and examination of potential health effects. The California sea lion, an apex predator with a foraging range of its primary female population within the major DDT industrial outfall [64], accumulates some of the highest levels of DDT of any species, with values exceeding 1 mg/g blubber wet weight [65]. A reflection of these high levels of DDT is the finding of modeling studies which indicate that sea lion carcasses were the primary DDT vector responsible for the Channel Island bald eagle demise [66]. The common DDT metabolite *p,p’*-DDE (1,1-*bis*-(4-chlorophenyl)-2,2-dichloroethene) has been shown in physiologically-based pharmacokinetic models to increase in concentration in the fetus during the course of development, reach a peak during lactation and decline after that prenatally exposed animal’s first successful pregnancy (Figure 11) [64,67]. 

**Figure 12:**
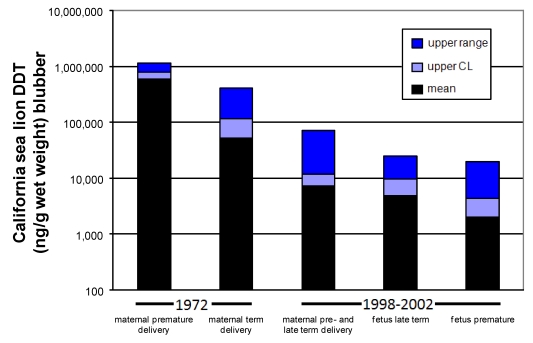
Amount of DDT found in maternal and fetal California sea lion blubber. Data from 1972 modified from [69] and from 1998–2002 is from [73].

Very high organochlorine levels, including not only DDT, but also polychlorinated biphenyls (PCBs), have been associated with reproductive failure of the California sea lion. These legacy contaminants of the post WWII industrial period co-occur in both the pregnant females and fetuses of this population. Six-fold higher concentrations of DDTs and PCBs were identified in females with aborted fetuses than females with term births during a 1970 reproductive toxicity event [68]. An experimental study of this sea lion population two years later showed the same trend, with eight-times higher DDT concentrations and four-times higher PCB concentrations in those females with aborted fetuses than females with normal term deliveries (Figure 12, two bars on left) [69]. However, the identification of two co-occurring pathogens, San Miguel Sea Lion Virus and *Leptospria pomona*, the later associated with reproductive failure in livestock, added a confounding factor that precluded implicating a role for PCBs or DDTs in the reproductive poisoning of these sea lions [69].

DDT binds the voltage gated sodium channel stabilizing an open state and inhibits inactivation [70]. Systemic exposure to DDT causes tonic-clonic seizures in humans [71] and a well characterized tremor syndrome in rats that includes hyperactivity, ataxia and paralysis [72]. However, adult animals are relatively resistant to DDT toxicity and the tremor syndrome is only seen at high doses. Susceptibility to DDT increases as age decreases, allowing for lower doses to impact young and developing animals. DDT is freely absorbed by the sea lion fetus in quantities proportional to lipid content with a majority of fetal DDT content present by midgestation [73], a time that corresponds to synaptogenesis [72]. Studies indicate neonatal mice during synaptogenesis retain 10-times higher levels of DDT in brain and are about 100-times more sensitive to DDT toxicity than adults [75,76]. 

*p,p’*-DDT exposure of mice during synaptogenesis leads to neurobehavioral alterations later in adult life [76]. At the cellular level, *p,p’*-DDT exposure at synaptogenesis alters development of muscarinic cholinergic pathways in the cerebral cortex [75]. The mechanism is consistent with an action of DDT on voltage gated sodium channels as evidenced by increased potassium-evoked release of acetylcholine in cortical slices [77]. Parallel studies with other environmental toxicants, including PCBs (polychlorinated biphenyls) and polybrominated diphenyl ethers (PBDEs), have established the general observation that a number of different agents disrupt neurodevelopment when administered during key milestones, which can potentiate the reaction to toxicants later in life [78,79,80]. PCBs and PBDEs share common actions to alter the thyroid hormone metabolism [81,82]. Thyroid hormone is essential for neuronal migration and synaptogenesis and may represent an additional target for developmental toxicity. Hence, the potential exists that two classes of environmental stressors, one persistent man-made contaminant(s) and the other an emerging neurotoxin produced by harmful algal blooms, may interact to cause greater harm in the marine environment. The neurotoxic consequences of interaction of mixtures chemical contaminants and toxins produced by harmful algal blooms is gaining increased attention in pinnipeds and cetaceans [74,83] and is described in greater detail in the next two sections.

## 7. Merging of an Algal Toxin and Persistent Pesticide at a Marine Sanctuary

These two classes of environmental stressors trends merge in a rich biological transition zone that supports diverse and abundant marine life. The shore line topography of the California Channel Islands National Marine Sanctuary and the redirection of offshore currents by a series of ridges and basins promote mixing of cool, nutrient rich waters from the north with warmer waters from the south. Female California sea lions inhabit rookeries at five of the Channel Islands year round and primiparous females in this population are vulnerable to reproductive poisoning. The Channel Islands are located at the crossroads of domoic acid producing harmful algal blooms and a residual seepage of a major discharge site of DDTs that persist in sediments of the Palo Verdes shelf. This unique location provides the spatial basis to investigate the coincidence of these two stressors on the sea lion population.

**Figure 13:**
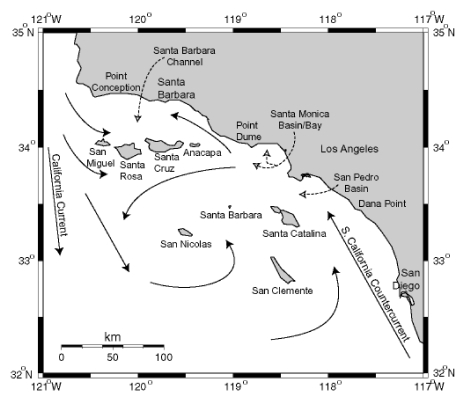
Circulation patterns in the Southern California Bight. Modified from [84], Copyright (1992) [85].

The California Channel Islands are eight islands on the northern edge of the geographically defined Southern California Bight. The region is geologically recognized as the Southern California (SC) Borderline, a complex grouping of basins and ridges at the merging of the Pacific and North American Plates. The four northerly islands comprise a ridge that rises to demark the Santa Barbara Channel (SBC) to the north, and the four southern islands are sea level ridges juxtaposed between six major basins southwest of Los Angeles. The northern islands receive the southern flow of the California Current (CC) which carries biologically productive, well-oxygenated waters southward from the Northern Pacific Ocean during spring and fall upwelling periods (Figure 13). The CC can split at the northern edge of San Miguel Island to flow nutrient rich waters into the SBC. This leads to exceptionally rich primary and secondary biotic production. The equatorial flow of the CC is displaced by the flow of the warmer South California Counter Current (SCCC) forming the massive Southern California Eddy which circulates around the southern Channel Islands. The SCCC reaches northward into the SBC and the mixing causes late spring eddies within the SBC. Local upwelling from the SBC eddies has been noted in 2002 and 2003 to intensify and prolong *P. australis* blooms associated with severe sea lion mortality events [86]. 

The world’s largest industrial outflow of DDT manufacturing was discharged through wastewater of southern Los Angeles County off the Palos Verdes Peninsula near the edge of the continental shelf descending into the San Pedro Basin. Elevated concentrations of the metabolite *p,p’*-DDE persist in the water column and sediments of the Palos Verdes Shelf, and remain biologically active in various fish species inhabiting the shelf area and northward to Santa Monica Bay. Sea lions of the Channel Islands have accumulated high levels of DDT, and modeling studies have indicated that sea lions with elevated DDT levels have likely received their input directly from the Palos Verdes Shelf [64]. 

The spatial distribution of domoic acid and DDT in the waters surrounding the Channel Islands place sea lions and other marine life associated with this rich transition zone at high risk for exposure of these two stressors. Yet as described in the next section the temporal accumulation of domoic acid and DDT may define the outcome of disease caused by these stressors. 

## 8. Exposure to DDT Enhances Domoic Acid Induced Seizures

Although DDT and domoic acid each cause neuroexcitation and as a result may cause an additive or synergistic effect when given at the same time, the outcome of California sea lions exposed to these stressors may depend on the timing of exposure and the timing of brain development of the sea lion. Exposure to DDT can be explained by toxicokinetics of maternal-fetal transfer across the placenta, providing it to the fetus throughout development [64,67]. The bulk of maternal DDT in sea lions is released from fat storage during a first pregnancy and as a result burdens the first born with the largest amounts of contaminants [87]. Exposure to domoic acid largely follows planktivorous fish contamination by seasonal upwelling patterns and local circulation patterns, with the largest poisoning events being in the late spring and extending into the summer. In the California sea lion, the late spring corresponds to an *in utero* period of functional brain maturation, while the summer period corresponds to either a prenatal period of embryonic diapause or postnatal nursing. This latter period is not thought to be at high risk for domoic acid poisoning since the diapause is a state of protected developmental suspension and transfer of toxin in contaminated milk by oral absorption is very low [74,88]. The temporal coincidence of DDT accumulation during fetal growth and domoic acid poisoning after functional maturation of the brain has been investigated in zebrafish, which has been increasingly utilized as a model to investigate development.

**Figure 14:**
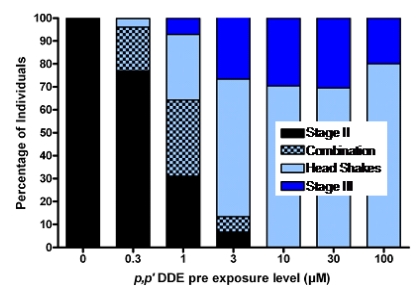
Percent of larval zebrafish responding with chemical induced seizures following embryonic exposure to concentrations of *p,p*’-DDE. Combination denotes those that exhibited both a definable Stage II seizure along with head shake behaviors. Data from [91].

Chemical induced seizures have been well studied in zebrafish and in spite of profound differences in brain structure, zebrafish share remarkably similar brain chemistry and convulsant induced seizure behaviors as found in both established models of laboratory rats [89,90] and observations in sea lions held at wildlife clinics [48]. Zebrafish are particularly well suited to exposure studies during development because of the rapid development period which can be directly observed in advance of pigmentation. Accordingly, zebrafish have been used as a first line test for the coincidental *p,p’*-DDE and domoic acid exposure scenario model for sea lions [91]. Zebrafish embryos have been exposed to *p,p’*-DDE, in much the same way as it transfers from mother to fetus. When functional brain maturation was complete the young were treated with either the model chemical convulsant pentylenetetrazole (PTZ) (a GABA-A antagonist) or domoic acid to mirror the likely timeframe of exposure which may be expected in sea lions, as poisoning frequently occurs at the end of pregnancy. The *p,p’*-DDE treated zebrafish embryos were asymptomatic upon completion of functional neurodevelopment, yet expressed increased sensitivity to PTZ or domoic acid induced seizures. They also expressed an unusual head shaking behavior (Figure 14) [92]. These results are consistent with experimental findings that DDT disrupts neurodevelopment when administered during key milestones, which potentiates the reaction to toxicants later in life [80]. 

The effective zebrafish dose calibrated to wet weight body concentration at the time of completed neurodevelopment, corresponds to the whole body *p,p’*-DDE of full term California sea lion fetuses, modeled with 1991 data based on the consumption of fish contaminated with 1,000 ng *p,p’*-DDE/wet weight. If this effective dose (4500 ng/g wet weight) is compared to actual wet weight body concentrations of full term California sea lions, it corresponds to the higher levels found in animals sampled in 1998–2002 and lower levels of animals sampled in 1972 (Figure 12). Collectively, these results provided a new perspective of how harmful algal blooms may react with *p,p*’-DDE which persists in the waters that surround the Channel Islands.

**Figure 15:**
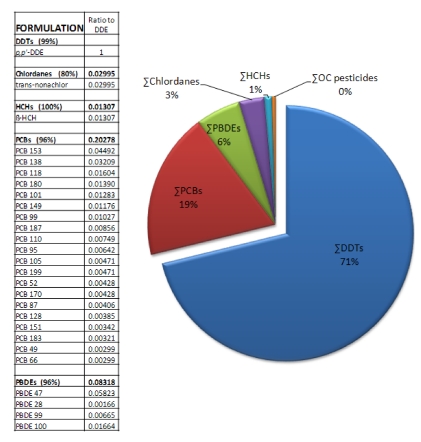
Sea lion contaminant formulation tested in zebrafish embryos [93]. Data on contaminant concentrations from [94].

Given the complex mixture of persistent contaminants such as PCBs, PBDEs, and persistent pesticides that co-occur with DDT in fetal sea lions, a compelling question is whether other dominant contaminant components of these groups contribute to the DDT effect to enhance domoic acid induced toxicity. To test this question, a subsequent study exposed zebrafish embryos to *p,p’*-DDE in the absence and presence of mixtures formulated to match the contaminant composition in fetal sea lion blubber (Figure 15) and then analyzed induced seizure behavior in the zebrafish after brain maturation [93]. These studies found that *p,p’*-DDE, in the presence of PCBs, hexachlorocyclohexane, chlordane and PBDEs at concentrations that co-occur in fetal sea lions, accounted for the full synergistic activity that leads to greater sensitivity to domoic acid seizures. Although PCBs and PBDEs at levels reported in fetal sea lions do not enhance domoic acid induced seizures, synergistic effect of persistent contaminants on the long term consequences of domoic acid poisoning (e.g., spatial memory impairment, recurrent seizures and atypical behaviors) require investigation. 

Developmental DDE poisoning, if it manifests in sea lions as observed in the zebrafish model, will predispose a subpopulation of “first born” sea lions to domoic acid toxicity. Sea lions with high DDE burden may also be more susceptible to domoic acid-induced seizures during fetal life, due to retention of domoic acid in the fetal compartment [56,59]. Whether fetal domoic acid poisoning progresses to neurologic disease in sea lions has not been documented; however, experimental studies in rodents clearly indicate that exposure to domoic acid during development has long term consequences. 

## 9. The Potential for a Fetal Origin of Epileptic Disease in Sea Lions

An interesting observation in analysis of epileptic syndrome in sea lions is the increased incidence of chronic disease in younger age sea lions. By contrast, acute poisoning predominates in adult females (Figure 10). The symptomatology of the epileptic syndrome differs between young and adult animals as well. One third of younger animals did not have seizures at admission and overall displayed lower grade symptoms of tremors, ataxia, or depression. The younger animals that died of epileptic syndrome had minimal or atypical neuropathological damage and not the hippocampal sclerosis and limbic system damage observed in adult sea lions. Although the difference in the response may be the younger age that the sea lions present with the disease, the poisoning during fetal life and expression of disease later in life is another possibility that has precedence from experimental investigation [74]. 

The developing brain responds to neurotoxic insult differently than the functionally mature brain. For example, kainic or domoic acid in late juvenile and adult rats elicit seizures which can in turn cause a brain damage syndrome similar to TLE. By contrast, these agents do not lead to neuron loss in the developing brain yet result in epileptic disease that can present a different array of symptoms. Utilization of this information in rats to consider neurological disease in young sea lions requires consideration for the differences in their development time lines. Sea lion neurodevelopment at birth is among the most advanced of all animals. Observation of newborn sea lions, brain growth and histomorphology are consistent with functional maturation of the nervous system that is largely complete at birth and on a comparable developmental time line to the rhesus macaque [74,95,96]. By contrast, rats are still in early stages of neurodevelopment at birth. Comparative scaling of neurodevelopment using brain growth indices and comparative histomorphology of the hippocampus, cerebrum, cerebellum and brain stem of sea lion brains at different stages of *in utero* development indicates that the laboratory model of a postnatal (day 0 to 21) rat would be closer developmentally to a fetal sea lion just past the middle of *in utero* development. [74]. 

**Figure 16:**
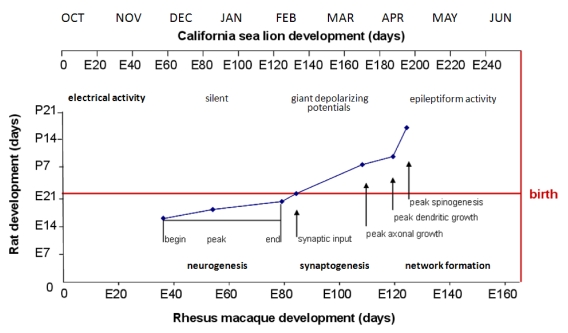
Correlation of Rat and Rhesus Monkey Neurodevelopment with Gestational Scaling to the California Sea Lion. Data from milestones of neurodevelopment in developing rat and monkey limbic brains is plotted from [97]. Scaling of fetal California sea lion development beginning with release of blastocyst from diapause projected to occur in the first week of October relative to rhesus macaque gestation is shown on top of chart. Overlay of hippocampal neurogenesis milestones is from reference [98]. Overlay of brain growth spurt is from reference [99]. The electrical activity time line is modified from rhesus macaque neurodevelopment [100] and scaled to the California sea lion.

Research data from mice and rats indicate that different patterns of seizures and associated behaviors result from domoic acid exposure during different neurological milestones of neurodevelopment. The first milestone, neurogenesis (which also includes neuromigration), occurs in the last third of gestation in rodents and is projected to occur in the second quarter of sea lion *in utero* development (Figure 16). The second development milestone, synaptogenesis occurs largely in the third week of postnatal life in rodents and is projected to occur in the third quarter of *in utero* development in sea lions. The third developmental milestone, network formation occurs later in postnatal life in rodents and is projected to occur in the final quarter of *in utero* development of the sea lion. 

Neurogenesis/neuromigration represents a time when the brain is electrically silent and proliferation of neurons occurs along primordial ventricles followed by migration to their correct brain region. The major pathways for glutamatergic and GABA-ergic transmitters are present but are functionally different than in the mature brain. Glutamatergic pathways at this stage are believed to guide the migration of neurons and GABA-ergic transmission is stimulatory and not inhibitory [101]. Domoic acid poisoning at this time likely leads to toxicity similar to neuronal migration disorder. This is evident from the following prenatal toxicity experiments in mice conducted on gestational day (GD) 13.

Toxicity studies in fetal mice during neurogenesis by exposure of subthreshold levels of domoic acid to dams at GD 13 revealed no observable effects in neonates at birth [102]. However, increasing irregularities in EEG recordings accompanied by a reduced threshold to domoic acid-induced seizures was observed at postnatal day (PND) 10 that intensified from mid to late postnatal life. Altered morphology of the hippocampus was not evident on the first postnatal day, yet began to appear midway through the postnatal period (PND 14), a time corresponding to the brain growth burst of synaptogenesis in mice. The altered morphology of the hippocampus was associated with a neurochemical basis for excitability, as indicated by an increased neurotransmitter ratio of glutamate:GABA in the brain and an increased density of kainic acid binding sites. The exposure to domoic acid at GD13 corresponds to the beginning of neurogenesis in the hippocampus and this suggests that toxic action on the proliferation or migration of neuroprogenitor cells may alter circuitry which promotes an excitatory (glutamatergic) over inhibitory (GABAerigic) balance in the hippocampus. Analogous effects also have been reported after *in utero* administration of the mitotic inhibitor methylazoxymethanol to rats consistent with an action to inhibit clonal expansion of neuroprogenitor cells [103]. 

As neurogenesis and migration reaches completion, the brain begins the second development milestone, synaptogenesis. The synapses are formed and the brain undergoes growth traditionally referred to as the brain growth spurt [99]. In rat and the rhesus macaque synaptogenesis represents a time of axonal and dendritic growth and the beginning of acquisition of dendritic spines. It also marks the first appearance of synaptic currents for both glutamate and GABA. The electrical activity is recorded as giant depolarizing potentials with electrochemical signals echoing the organization of synaptic contacts [101]. Domoic acid poisoning at this time leads to focal seizures that do not appear to spread to the limbic system. This is evident from postnatal toxicity experiments as conducted in rats during postnatal days 8–14 [104].

The long term consequences of titration of domoic acid during synaptogenesis (PND 8–14) occur later in life and have been investigated in both mature (PND 120) and aged (PND 510) adults. These consequences have been elucidated in a cohesive series of studies from Doucette and Tasker, forming the basis for a developmental model for epileptic disease as introduced in an earlier section [36,37,105,106,107,108,109,110]. Behavioral effects in the animals as adults include altered nicotine place conditioning and reversal learning of spatial tasks. Biochemical and cellular alteration in the hippocampus include elevated brain-derived neurotrophic factor expression, reduced number of neurons in the dentate hilus, increased mossy fiber sprouting in the dentate gyrus and areas CA3 and CA1, along with elevated trkB receptor expression. These animals also show altered responses to the presentation of novel stimuli and reward with reduced prepulse inhibition to auditory intensity. Observation of animals in tests that present novel and spatial information (Morris water maze, Novel water maze, Open field) revealed a unique behavioral syndrome similar to class 2 seizures of the modified Racine scale [111]. 

Network formation is a developmental milestone that marks the transition of brain organization to functional maturation [112]. This milestone is initiated with the expression of dendritic spines which mature with increasing synaptic contact. White matter expands with axons populating pathways, gyri appears from expanding surface area of axons and dendritic aborization together with segregation of the interior into to lamaella and layers. In rat and the rhesus macaque synaptogenesis represents a landmark change in neurotransmitter function [35]. Corresponding with expression of chloride exporter KCC2, the reversal potential for chloride changes and GABA is no longer excitatory. GABA becomes the major inhibitory pathway, working in counterpoint to glutamatergic transmission. 

The neuroexcitiatory effects and resulting neuropathology for domoic acid in the functionally mature brain has been well described in humans, rodents and sea lions. As discussed earlier the long term consequences of domoic acid induced damage have been reported as temporal lobe epilepsy in a human case study and a similar syndrome over one hundred sea lions. More recently, domoic acid induced status epilepticus has been demonstrated to develop after a latent period of weeks to a progressively worsening state of recurrent spontaneous seizure in rats. It is interesting that recurrent spontaneous seizures have been proposed to reverse the inhibitory-to-excitatory shift of GABA, returning neurons to immature properties that are no longer able to restrain neuroexcitation [113].

A possible fetal origin to epileptic disease in the sea lion is important to recognize because of the greater sensitivity of fetal animals to domoic acid poisoning. Pharmacokinetic mechanisms may include sustained action of domoic due to poor elimination of the toxin from the fetus. Pharmacodyamic mechanisms may include reduced threshold for seizures resulting from DDT disruption of brain development. A molecular target for DDT is the voltage-gated sodium channels and a role for these channels in the fetal origin of epilepsy is supported by documentation that mutation in the alpha-1 subunit of the voltage gated sodium channel leads to febrile seizures and epilepsy [114]. Poisoning in fetal life might represent the only time that domoic acid poisoning may progress to epileptic disease in cetaceans. Adult cetaceans are unlikely to survive status epilepticus, yet a fetus may survive poisoning when the exposure is below threshold for symptoms in the mother. Because each of the three milestones of neurodevelopment (neurogenesis, synaptogenesis and network formation) are projected to occur during *in utero* life of the sea lion (and likely cetaceans as well), a full range of patterns of seizures and associated behaviors can be expected to be observed in young animals. 

## 10. Calendric Projection of Outcome to Fetal Poisoning

Based upon experimental data in laboratory animals, domoic acid poisoning in sea lions may result in the expression of an epileptic syndrome of three potential age-dependent variations. Because fetal development of populations of sea lions is believed to occur in synchrony as a result of diel release of embryonic diapause [115], age-dependent variations in epileptic syndrome should be correlated to the time of year for a given poisoning event. 

**Figure 17:**
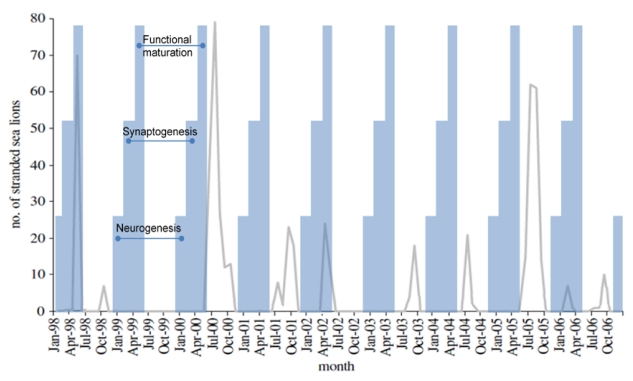
Correlation of sea lion stranding data with projected times of *in utero* neurodevelopment. Figure from [55] is modified with data from [74].

Poisoning during neurogenesis is anticipated to lead to a neuronal migration toxicity expressing a disease state with characteristics similar to that described by Dakshinamurti *et al*. [102], Levin *et al*. [116] and Tanemura [117]. Animals would mature asymptomatically with an increased excitability of the brain to stimulatory agents, such as exposure to domoic acid later in life, and a decreased gender‑based cognitive reserve and alteration in emotional behaviors. The developmental timeline would correspond in the sea lion to exposure during December and January, a time when poisoning events are not usually recorded (Figure 17). 

Poisoning during synaptogenesis is anticipated to lead to animals developing with an additional spectrum of gender-based learning deficits and increased emotionality behaviors. The latter would be associated with low grade seizure behaviors, more likely the result of novelty induced seizure response to stressful situations [37,118]. These animals would likely express a disease state with characteristics similar to those described by Doucette *et al*. [36,119]. This time of development for sea lions would correspond to poisoning between February and April, such as the poisoning event described in the early spring of 2006 (Figure 17). 

Poisoning after functional maturation of the brain would lead to limbic seizures and status epilepticus with characteristics similar to that described by Muha and Ramsdell [32] in late juvenile rats. The developmental timeline would correspond to *in utero* sea lion poisoning during the months of May and June, such as the poisoning events described in late spring of 1998 and 2002 (Figure 17). This syndrome could also initiate with domoic acid poisoning in animals of any age after birth; however poor gut absorption of domoic acid in milk places nursing likely lowers the risk in postnatal animals [74]. 

## 11. Summary

The view of the scientific community on domoic acid has changed considerably since the 1987 ASP event. Domoic acid is no longer just a shellfish toxin, because it is broadly distributed throughout various levels of the food web. Highly efficient vectors such as the northern anchovy can rapidly magnify exposure levels sufficiently to severely poison thousands of marine animals, compared to the poisoning of only a few high risk individuals in the Canadian ASP event. The progression of domoic acid induced status epilepticus to chronic epileptic disease changes the way the impacts of harmful algal bloom events are viewed in the marine environment. These events poison multiple species and neurological disease can appear months after an acute poisoning event and may also affect a second generation with disease appearing years after fetal exposure. Domoic acid poisoning of sea lions has brought the kainic acid model for epilepsy to life. Its significance to understand epileptic disease originating in fetal life and its value in uncovering difficult to predict synergistic interactions with environmental contaminants reveals potentially remarkable insights from a mere folk remedy to control flies.
